# Adjustable Prosthetic Sockets Are a Potential Solution to Skin Breakdown for Individuals with Lower Limb Loss: A Case Report

**DOI:** 10.3390/reports9020125

**Published:** 2026-04-20

**Authors:** Jessica Kenia, Jim Marschalek, Timothy Dillingham

**Affiliations:** 1 Department of Physical Medicine and Rehabilitation, University of Pennsylvania School of Medicine, Philadelphia, PA 19146, USA; 2 Advanced Design Concepts, Oconomowoc, WI 53066, USA

**Keywords:** amputation, limb loss, wound healing, ulcer, skin breakdown

## Abstract

**Background and Clinical Significance**: Conventional hard sockets are reported to result in skin breakdown for almost half of transtibial prosthesis users. Adjustable sockets have been developed to better accommodate residual limb shape and volume changes. They have demonstrated optimal skin health in prospective adult clinical studies. **Case Presentation**: We present the case of a 57-year-old male with a transtibial amputation who enrolled in a research study at the University of Pennsylvania. In the year before enrollment, he experienced frequent, near-constant skin breakdown of the distal residual limb at the anterior tibia due to limb volume fluctuations and excessive pressure from a conventional hard socket and was frequently unable to use his socket due to skin breakdown. The subject was fit with an adjustable, immediate fit transtibial prosthesis (iFIT Prosthetics^®^). After a two-week home trial, he rated the adjustable prosthesis 62 out of 70 on an adapted Prosthetic Evaluation Questionnaire, compared with a score of 20 for his conventional prosthesis. Due to improved comfort, he discontinued the use of his conventional device. The subject was followed for over one year and wore the adjustable prosthesis exclusively without a recurrence of skin breakdown. Residual limb volume changes commonly lead to poor socket fit and skin irritation in conventionally fabricated hard sockets, often progressing to skin breakdown. In individuals with diabetes, wound healing can be prolonged and functionally limiting. In this case, an adjustable prosthesis successfully eliminated anterior tibial skin breakdown in a subject predisposed to this injury when using conventional hard sockets. **Conclusions**: Adjustable sockets can prevent skin breakdown in individuals with transtibial limb loss.

## 1. Introduction and Clinical Significance

Conventional prosthetic socket fabrication techniques have largely remained unchanged for more than five decades. These methods are typically labor-intensive, require multiple clinical visits, and result in rigid sockets with a limited capacity for modifications [1,2]. As a result, conventional sockets often fail to accommodate the dynamic nature of residual limb volume changes, particularly during the first year following amputation [3].

During this post-amputation period, substantial reductions in residual limb volume commonly occur as edema dissipates, frequently resulting in a poorly fitting prosthetic socket [3,4]. Multiple sockets may need to be fabricated to accommodate these changes. Individuals with comorbidities such as diabetes mellitus, cardiovascular disease, or renal disease may experience additional daily fluctuations in limb volume due to vascular and fluid balance changes [5,6]. These factors contribute to discomfort, instability, and skin irritation and breakdown within the socket. As a result, dissatisfaction with prosthetic devices is a commonly reported issue, with 44% to upwards of 63% of surveyed prosthesis users reporting dissatisfaction related to comfort and fit [7,8].

An improper socket fit increases the shear stress and pressure on the residual limb, which can compromise skin integrity and lead to pain, ulceration, or infection. Skin breakdown associated with conventional rigid sockets is a frequent complication, reported in 36–40.7% of individuals with lower limb prostheses [9,10]. When skin breakdown occurs, patients are often instructed to limit or discontinue prosthesis use to allow for wound healing, significantly restricting mobility and independence and negatively impacting quality of life.

In response to these limitations, adjustable prosthetic sockets have emerged as an alternative approach designed to accommodate residual limb volume fluctuations. Unlike conventional rigid sockets, adjustable systems allow for user-driven socket fit modifications, enabling changes in circumferential compression without socket replacement. This adaptability has the potential to reduce shear forces, improve suspension, and maintain consistent contact during periods of limb volume instability. In multiple clinical trials comparing a recently developed adjustable socket to conventional prosthetic sockets, skin breakdown was eliminated [11,12,13].

Many prior studies have focused on patient comfort, overall satisfaction, and short-term results. The present case study followed a patient with friable skin and recurrent tibial skin breakdown who experienced complete resolution of chronic lesions following transition to an adjustable socket. This case highlights the potential for adjustable socket technology not only to improve comfort, but to fully resolve persistent skin complications associated with conventional rigid sockets.

## 2. Case Presentation

This case involved a 57-year-old male with a unilateral transtibial amputation who enrolled in an IRB ethics committee-approved (#855019) study at the University of Pennsylvania, conducted in accordance with the Declaration of Helsinki. This case report focuses on this participant, who is part of an ongoing single-group prospective study designed to evaluate a newly designed adjustable transtibial prosthesis compared with a conventional prosthesis. Data collection for the parent study is ongoing, and the present report highlights the clinical outcomes of this individual.

The participant reported limb loss due to diabetes approximately one year prior to the initial visit and had been using a prosthesis for 10 months. His diabetes was managed with medication under the care of his primary physician. At the initial assessment, he was classified as a K3-level ambulator. The PI assessed that the participant had good hand function and cognitive ability and was able to follow instructions and understand how the device functioned.

In the year preceding study enrollment, the patient experienced frequent and nearly continuous skin breakdown localized to the distal residual limb and anterior tibia. These complications were attributed to significant residual limb volume fluctuations and excessive pressures generated by a conventionally fabricated rigid socket. The patient reported substantial frustration with prosthesis use, noting that recurrent skin breakdown frequently confined him to his home while awaiting wound healing. His conventional socket wear time was reported to be 9+ hours per day; however, he was unable to use his prosthesis for several days once his skin broke down. This interruption in prosthesis use significantly limited his ability to perform routine daily activities. He indicated that he was unable to obtain a replacement socket due to insurance limitations. Despite using socks to replace the volume lost, he was still unable to achieve a well-fitting socket.

At baseline, the subject evaluated his conventional prosthesis using a questionnaire adapted from the Prosthetic Evaluation Questionnaire (PEQ) [14]. The adapted instrument consisted of 14 items scored on a five-point scale, yielding a maximum score of 70 points. This survey was modified from its original form due to length and the desire to focus on socket fit and comfort rather than overall quality of life, as the original survey intended. While this adapted questionnaire has not been validated, it has been utilized for assessing the prosthesis itself by the research team in the evaluation of similar prosthetic devices [11,12,13]. The subject also completed a 3D walking gait analysis using an eight-camera Vicon motion analysis system (version 1.8.5, Oxford, UK). The gait analysis was completed to assess temporal measures of the gait. Lastly, peak pressures were identified in the socket using Fujifilm (Tokyo, Japan) Prescale film (Extreme Low), which captures 7 to 28 psi. The film changes color intensity according to the peak (maximum) amount of pressure applied and is then graded to determine the peak pressure. The Fujifilm was placed in five locations on the patient’s liner: the proximal tibia, distal tibia, and medial, lateral, and posterior aspects of the limb. The participant donned their socket and proceeded to walk for the gait analysis, which consisted of six trials walking on a 10 m walkway. The film was then removed and evaluated. The participant had his skin evaluated before fitting the prosthesis to ensure all wounds were healed. These outcome measures were repeated during the two-week follow-up on the adjustable prosthesis.

**Figure 1:**
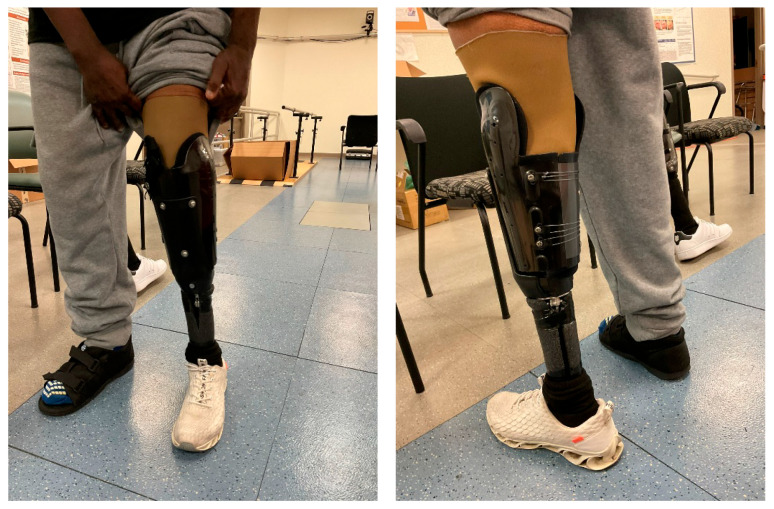
The iFIT adjustable prosthesis (Transtibial Advanced Closure—TTAC model), which utilizes supracondylar support and a pulley mechanism for adjustments.

Following the initial evaluation, he was fit with an adjustable, immediate fit transtibial prosthesis (iFIT Prosthetics^®^, Oconomowoc, WI, USA) during a single clinical session lasting approximately two hours (Figure 1).

**Figure 2:**
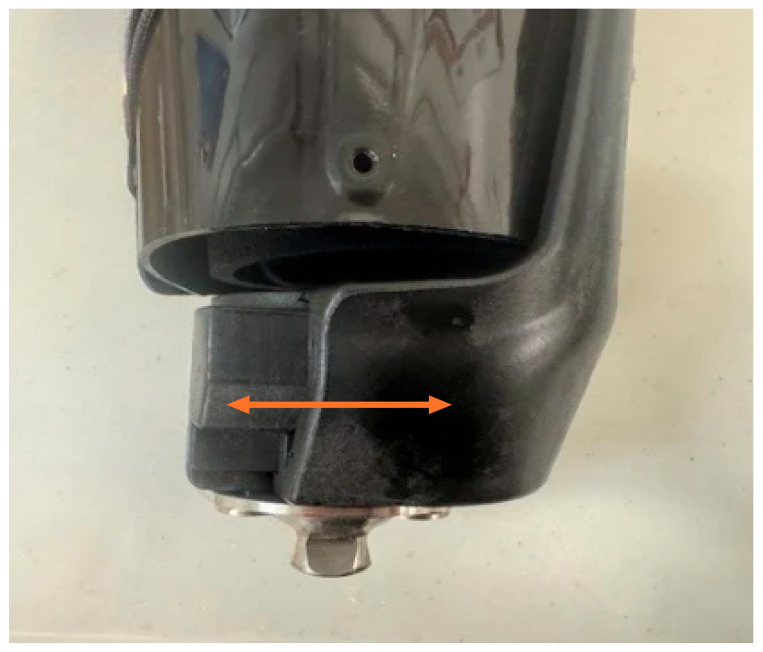
The base of the cup extends forward and backward 1.5 cm to accommodate varying limb circumferences.

**Figure 3:**
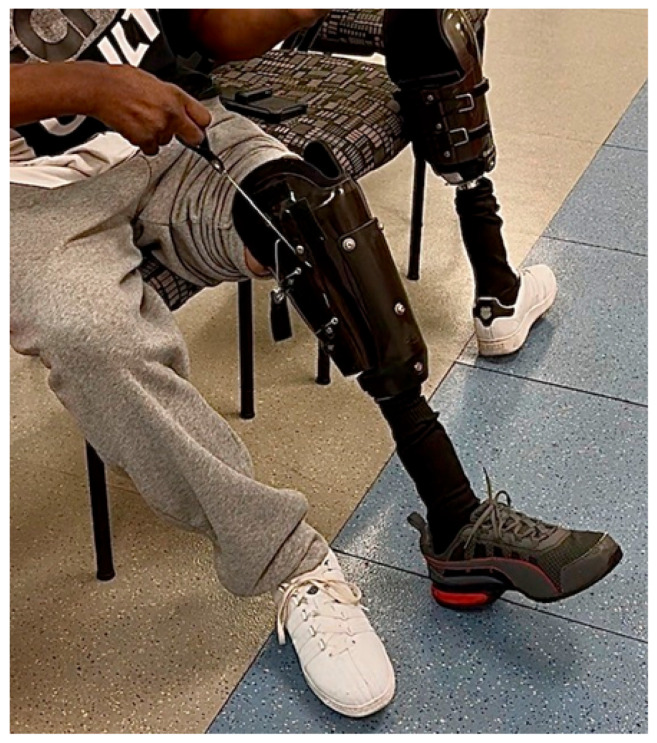
The subject demonstrates how to tighten and adjust the circumference of the prosthesis by pulling the attached cord.

The adjustable prosthesis used is a modular system that utilizes high-strength, lightweight proprietary material and does not require casting, molding, or check sockets. It is fit using hand tools in a single visit in a couple of hours. The system features an adjustable base cup that was adjusted by the clinician to fit the patient’s distal circumference during the initial visit (Figure 2). The socket is available in three base sizes based on circumferential measurements (Pediatric, Standard, or Wide) and in three lengths based on the residual limb length measurement (Short, Medium, or Tall). The system is integrated with a silicone pin suspension liner worn by the patient. It is tightened around the residual limb using an internal pulley mechanism, which can then be readily adjusted anytime by the wearer (Figure 3).

After fitting, the patient demonstrated stable ambulation and the ability to independently adjust, don, and doff the prosthesis. The patient was then instructed to wear the prosthesis during a two-week home trial period. At the conclusion of the two-week trial, the patient re-completed the adapted PEQ. The adjustable prosthesis received a score of 62 out of 70, compared with a baseline score of 20 for his conventional prosthesis. Each individual question is shown in Table 1. His gait analysis revealed a faster cadence and walking speed when wearing the adjustable prosthesis (Table 2). His peak pressure measurement decreased the proximal tibial area, lateral and posterior areas of the limb (Table 3).

**Figure 4:**
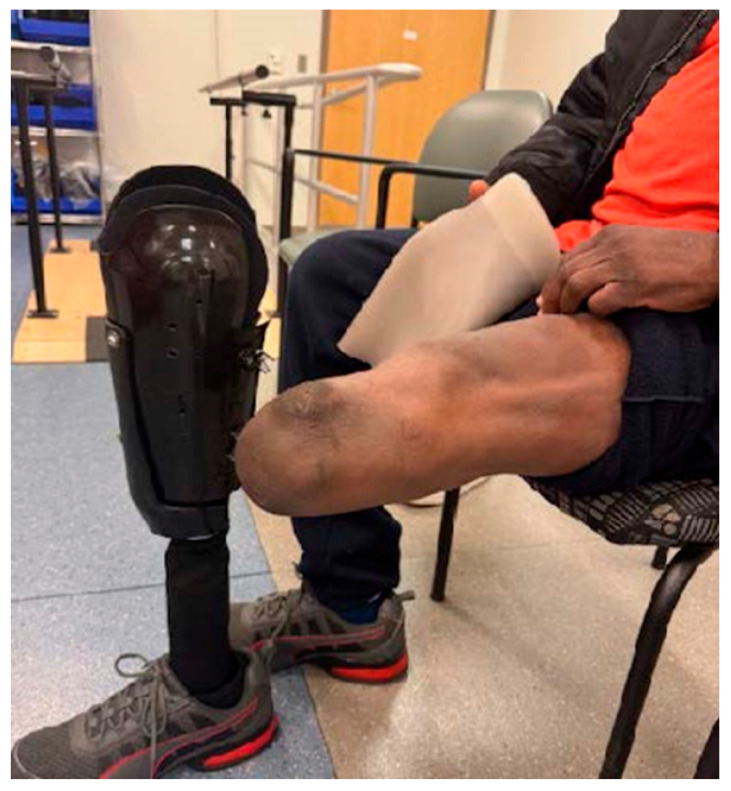
The subject presents his limb free of any skin irritation or breakdown during his two-week evaluation. The PI verified the skin on his anterior tibia, site of previous breakdown, was in-tact and free of redness or signs of irritation.

He reported wearing the prosthesis for 9 or more hours per day without interruption. Due to the marked improvement in comfort and function, the patient voluntarily discontinued the use of his conventional prosthesis. He indicated that his limb was fully healed and did not acquire any skin breakdown while using the adjustable prosthesis (Figure 4).

The subject was followed longitudinally for over one year, and attended follow-up visits every two months to inspect his skin and the prosthesis. Throughout the follow-up period, he indicated he had no episodes of skin breakdown, maintained full prosthetic use, and experienced no activity restrictions related to residual limb skin integrity. He reported that he was able to participate in more activities outside of the home than with his previous socket and was walking more during the day. The subject described his daily activities including playing pool, playing drums, and engaging with his grandchildren. The adjustable socket felt more “secure” on his limb, and he had no issues tightening or adjusting it. The subject also reported that he preferred the adjustable socket to the conventional one and would continue to utilize this type of system.

## 3. Discussion

This case study describes a diabetic participant fitted with an adjustable transtibial prosthesis who experienced resolution of chronic skin breakdown. His gait analysis revealed a faster walking gait and cadence while wearing the adjustable prosthesis. Walking speed is a widely accepted indicator of functional mobility and overall health, with faster gait speeds associated with improved balance, functional performance, and survival [15,16,17]. Lower peak socket pressures were observed in selected regions of the participant’s residual limb. Although no normative pressure thresholds were available for comparison, lower peak interface pressures have been associated with reduced incidence of skin irritation and tissue breakdown [18,19].

Residual limb volume fluctuations remain one of the most persistent and clinically significant challenges in lower limb prosthetic management [5]. Conventional rigid sockets are fabricated to a fixed shape and cannot easily accommodate the dynamic changes in limb volume that occur throughout the day or over longer periods [1]. Even small fluctuations can disrupt the pressure distribution within the socket, leading to elevated shear forces, pistoning, and focal pressure peaks [5]. Over time, these mechanical stresses compromise skin integrity and contribute to pain, ulceration, and reduced prosthesis use. The patient described in this case exemplifies the cumulative impact of these issues; despite sock management, he was unable to maintain a stable fit and experienced near-continuous skin breakdown.

These challenges are particularly pronounced in individuals such as the participant with diabetes mellitus or those with vascular disease and renal impairment, where limb volume may fluctuate due to fluid shifts [20]. For these populations, even minor skin lesions can progress to more serious complications, prolonging rehabilitation and limiting mobility [20]. Insurance restrictions and lengthy fabrication timelines for conventional sockets further compound the problem by limiting access to timely socket replacement, effectively forcing patients to continue using ill-fitting prostheses [21].

Adjustable socket systems are designed to address these limitations by allowing for real-time socket fit modifications by the patient without removing the prosthesis. By accommodating daily and long-term volume fluctuations, these systems may reduce mechanical stress at the limb–socket interface and help prevent the development of skin injury. The outcomes observed in this case are consistent with prior studies demonstrating improved comfort, stability, and user satisfaction with adjustable prosthetic systems [11,12,13]. These prospective studies also reported better fit and reduced adverse skin outcomes associated with adjustable prosthetic designs.

An adjustable transfemoral interface was found to improve subjective and performance measures 19% to 37% over conventional sockets during baseline conditions [22]. These authors also report greater improvements in all subjective and performance measures 22% to 93%, during volume loss conditions [22]. Our findings are consistent with prior literature reviews on adjustable prosthetic sockets, which report improvements in fit, comfort, and pressure distribution [23,24]. There is a growing recognition of adjustable designs as a clinically valuable approach to managing residual limb volume fluctuations. The patient in this case extends those findings by demonstrating complete resolution of recurrent skin breakdown over a prolonged period (>1 year), suggesting that adjustable socket technology may play a critical role in preventing chronic dermatologic complications

One limitation of adjustable sockets is that many prosthetists have not yet adopted this technology. As a relatively new approach, it often requires clinicians to complete brief training to effectively implement adjustable sockets in patient care. Additionally, certain populations may not be ideal candidates for adjustable prosthetic systems, including individuals with limited hand function or those who lack the cognitive ability to independently operate the adjustment mechanisms.

This case underscores the clinical value of adjustable socket technology as a practical, scalable potential strategy for preventing skin complications and maintaining prosthesis use. Wider adoption may reduce the burden of skin breakdown, enhance mobility, and improve long-term outcomes for individuals with lower limb loss.

## 4. Conclusions

This case study demonstrates that a novel adjustable transtibial prosthesis effectively prevented recurrent skin breakdown in a patient who previously experienced frequent skin lesions with a conventional rigid socket. Adjustable socket technology represents a promising intervention for individuals at high risk for skin complications due to residual limb volume fluctuations. The broader adoption of adjustable sockets may improve comfort, prosthesis satisfaction, and long-term skin health in vulnerable populations.

## Figures and Tables

**Table 1:** Adapted Prosthetic Evaluation Questionnaire (PEQ) Ratings for a Single Case (5-Point Scale).

Domain	Conventional Prosthesis	Adjustable Prosthesis
Fit and alignment	1	5
Comfort while standing	2	5
Comfort while walking	1	5
Comfort while walking long distances	1	4
Prosthesis weight	1	5
Stability while standing	3	5
Stability while walking	2	5
Ease of donning and doffing	1	4
Temperature of limb	2	4
Stair ascent and descent	3	5
Accommodation of residual limb volume changes	2	5
Overall satisfaction	1	5

Note. Ratings reflect responses from a single participant. The conventional prosthesis was evaluated during the initial intake and fitting visit. The adjustable prosthesis was evaluated at the two-week follow-up visit. Ratings were based on a 5-point Likert scale, with higher scores indicating greater satisfaction.

**Table 2:** Temporal–Spatial Gait Parameters for a Single Case Using Conventional and Adjustable Prostheses.

Parameter	Conventional Prosthesis		Adjustable Prosthesis	
	Left	Right	Left	Right
Cadence (steps/min)	89.0 ± 3.08	88.6 ± 4.23	96.8 ± 4.45	95.9 ± 2.78
Double support (s)	0.48 ± 0.041	0.48 ± 0.061	0.40 ± 0.026	0.41 ± 0.038
Single support (s)	0.42 ± 0.016	0.46 ± 0.036	0.41 ± 0.018	0.44 ± 0.045
Limp index	0.97 ± 0.033	1.04 ± 0.05	0.97 ± 0.039	1.05 ± 0.073
Step length (m)	0.45 ± 0.046	0.46 ± 0.051	0.58 ± 0.026	0.54 ± 0.017
Step time (s)	0.69 ± 0.051	0.66 ± 0.036	0.63 ± 0.036	0.62 ± 0.041
Stride length (m)	0.93 ± 0.073	0.93 ± 0.061	1.13 ± 0.025	1.12 ± 0.024
Stride time (s)	1.35 ± 0.047	1.36 ± 0.064	1.24 ± 0.058	1.25 ± 0.037
Walking speed (m/s)	0.69 ± 0.065	0.69 ± 0.065	0.91 ± 0.039	0.89 ± 0.034

Note. These values represent the mean and standard deviations of three walking trials in each condition. The prosthesis was worn on the left limb; the right limb represents the intact limb.

**Table 3:** Peak intra-socket pressure comparison between conventional and adjustable prosthetic sockets.

Socket Region	Conventional Prosthesis (psi)	Adjustable Prosthesis (psi)	Δ Pressure (psi)
Proximal Tibia	28.0	26.5	−1.5
Distal Tibia	28.0	28.0	0.0
Medial	26.5	26.5	0.0
Lateral	26.5	25.0	−1.5
Posterior	28.0	22.5	−5.5

Note: Peak intrasocket pressures during walking were measured using Fujifilm Prescale film at five anatomical socket regions. Values represent the maximum pressure recorded in each region rather than the average sustained pressure.

## Data Availability

The original contributions presented in this study are included in the article. Further inquiries can be directed to the corresponding author.
